# Patients’ experiences of living with medically unexplained symptoms (MUS): a qualitative study

**DOI:** 10.1186/s12875-018-0709-6

**Published:** 2018-02-02

**Authors:** Agnieszka Sowińska, Sławomir Czachowski

**Affiliations:** 1 Department of English, Nicolaus Copernicus University, ul. W. Bojarskiego 1, 87-100 Toruń, Poland; 2 Department of Psychology and Centre for Modern Interdisciplinary Technologies, Nicolaus Copernicus University, ul. Gagarina 39, 87-100 Toruń, Poland

**Keywords:** Medically unexplained symptoms, Patients’ experiences and expectations, Qualitative research, Poland

## Abstract

**Background:**

Patients with medically unexplained symptoms (MUS) are common in primary care, and pose a communicative and therapeutic challenge to GPs. Although much has been written about GPs’ frustration and difficulties while dealing with these patients, research presenting the patients’ perspectives on MUS still seems to be scarce. Existing studies have demonstrated the patients’ desire to make sense of symptoms, addressed the necessity for appropriate and acceptable explanation of MUS, and revealed stigmatization of patients with symptoms of mental origin. Treatment in primary care should focus on the patient’s most essential needs and concerns. The objective of this paper is to explore Polish patients’ perspectives on living with MUS.

**Methods:**

A qualitative content analysis of 20 filmed, semi-structured interviews with patients presenting MUS (8 men and 12 women, aged 18 to 57) was conducted. All patients were diagnosed with distinctive somatoform disorders (F45), and presented the symptoms for at least 2 years. The interviews were transcribed verbatim and analysed independently by two researchers.

**Results:**

Four major themes emerged: (1) experiences of symptoms; (2) explanations for symptoms; (3) coping; (4) expectations about healthcare. Within the first theme, the patients identified the following sub-themes: persistence of symptoms or variability, and negative emotions. Patients who observed that their symptoms had changed over time were better disposed to accept the existence of a relationship between the symptoms and the mind. The second theme embraced the following sub-themes: (1) personal explanations; (2) social explanations; (3) somatic explanations. The most effective coping strategies the patients mentioned included: the rationalization of the symptoms, self-development and ignoring the symptoms. The majority of our respondents had no expectations from the healthcare system, and stated they did not use medical services; instead, they admitted to visiting psychologists or psychiatrists privately.

**Conclusion:**

Patients with MUS have their own experiences of illness. They undertake attempts to interpret their symptoms and learn to live with them. The role of the GP in this process is significant, especially when access to psychological help is restricted. Management of patients with MUS in the Polish healthcare system can be improved, if access to psychologists and psychotherapists is facilitated and increased financial resources are allocated for primary care. Patients with MUS can benefit from a video/filmed consultation with a follow-up analysis with their GP.

## Background

Patients with medically unexplained symptoms (MUS) are extremely common in primary care [1]. They account for approximately 25% of primary care consultations [2] and up to 50% of secondary care outpatient appointments [3], whereas around 3–10% of adult GP consulters are estimated to have persistent or recurring MUS [4, 5]. Diagnosing, explaining, and managing MUS in primary care often poses a challenge. Much of the frustration also relates to difficulties with definition and classification [6–8]. Medically unexplained symptoms or functional disorders refer to “conditions where the patient complains of physical symptoms that cause excessive worry or discomfort or lead the patient to seek treatment, but for which no adequate organ pathology or patho-physiological basis can be found” [9]. In other words, when symptoms persist but cannot be attributed to disease following clinical investigation, they are generally considered medically unexplained symptoms (MUS) [6]. In psychiatry, particular constellations of MUS are classified as somatoform disorders (F45) in line with the International Classification of Diseases (ICD-10) [10]. Somatoform disorders may include, for example, somatization disorder, pertaining to patients with MUS that encompasses all bodily systems [8].

Existing scientific theories explaining the outbreak of medically unexplained symptoms such as, for example, somatization, which assumes the presence of bodily symptoms as indirect markers of psychological distress, or somatosensory amplification, explaining MUS as a result of stress-related physiological arousal, are neither fully satisfactory nor definitive [6, 8, 11].

Patients with MUS are believed to have difficulty maintaining communication, their illness narratives are often chaotic, lacking a clear sequence of chronological events representing development of symptoms [12, 13]; they signal unrealistic expectations, and expect frequent referrals to secondary care and frequent diagnostic tests. They are not only a communicative and therapeutic challenge, but they are also a heavy financial burden [14, 15]. Doctors often label them as ‘heartsink’ patients, that is the patients that evoke negative emotions or, to put it simply, “cause the heart to sink” [16].

Though much has been written about GPs’ frustration and difficulties while dealing with these patients, existing studies that present patient perspectives on MUS have repeatedly shown that patients simply wish to make sense of their symptoms [6, 13, 17–19]. Research on the patients’ experiences often focuses on the relevance of patient explanations of MUS, and factors contributing to improvement [20–22]. Discriminating attitudes and stigmatization of patients with symptoms of mental origin have also been revealed in research [23, 24].

Treatment in primary care should focus on the patient’s most essential needs and concerns. In Poland, where family medicine is relatively new, there are no guidelines for management of patients with MUS, and Polish GPs are not always responsive to the patients’ needs for reassurance and empathy [25, 26].

This study is part of a larger research project on the role of MUS patients’ verbal and non-verbal behaviour in doctor-patient communication in a Polish primary care setting. While Polish GPs’ experiences of MUS have already been described [27], the objective of this study was to understand and explore patients’ perspectives on living with MUS. Specifically, the study aimed to answer the following research question: what are Polish patients’ experiences of living with MUS?

## Methods

### Design, setting and characteristics of the sample

A qualitative content analysis of 20 filmed, semi-structured interviews with patients presenting MUS was performed. The interviews took place in a doctor’s surgery at the Centre for Modern Interdisciplinary Technologies at Nicolaus Copernicus University in Torun, Poland, between February 2015 and December 2015. A purposive sample of 20 patients (8 men and 12 women, aged 18 to 57) was first recruited through a single general practice, which provides medical services to 4000 patients [28]. The mean age of the interviewees was 37.4 years. The patients who were diagnosed with F45, that is, psychosomatic or somatoform disorders according to ICD-10, and who had complained about the symptoms for at least 2 years, were invited to participate in the study. Twenty-one patients were contacted by phone or directly by their GP, and only one refused to be filmed. All patients were also asked to fill in the Four-Dimensional Symptom Questionnaire (4 DSQ) - a test that is used to evaluate and measure the four most common mental disorders in general practice: distress, depression, anxiety, and somatization - and include the related symptoms that had occurred over the last week [29]. Some patients scored low on the somatization dimension, which indicates that they did not present any symptoms in the week when the interview was conducted - which, in turn, may suggest they coped well with the symptoms. Although the 4DSQ test results were relevant for the subsequent stages of the research project, which selected patients with high somatization to explore their behaviour, they did not affect the present study and qualitative content analysis of the interviews. The patients gave their written informed consent to participate in the study and to be filmed, on condition that they would remain fully anonymous. They wanted to remain anonymous in the published data, and to be interviewed by the doctor they had known well and whom they trusted, refusing to be interviewed by anyone else. There were 14 patients with higher education, six with secondary education, and some patients were highly educated and held prestigious jobs. In the interviews, they often revealed deeply personal and secret details from their life, which were triggers of MUS narratives. Their major symptoms included: headaches, backpain, muscle pain, chest pain, abdominal pain, blackouts, oversweating, palpitations, blurred vision, fatigue, shortness of breath and dizziness. According to available medical records, three patients suffered from chronic conditions: chronic sinusitis, moderate arterial hypertension and varicose veins.

The interviews were conducted by the second author (SC), who was the only interviewing researcher and the family doctor of the majority of the patients, according to the topic guide derived from the literature on MUS [30], shown in Table 1. To ensure the patients’ understanding, the interview questions were pilot tested with two patients with MUS, in 2014. Ethical approval of the study’s research plan, including pilot testing, was granted by the Bioethical Commission of the Kuyavian-Pomeranian Doctors Chamber in Torun, Poland, in September 2014.

**Table 1:** Topic guide

Somatic dimension
Could you describe your symptoms? Do you remember when they started? Are the symptoms the same or do they change with time?
Cognitive dimension
What do you think causes the symptoms? How can you explain the symptoms? What do you think you can do to influence the symptoms?
Emotional dimension
What do you feel as a result of the symptoms? How did you react when they first appeared? Describe your emotions.
Behavioral dimension
What influence do the symptoms have on your life? Do you seek medical help? How do you cope with the symptoms?
Social dimension
What are the consequences of the symptoms on people around you? What effect do the symptoms have on your personal and professional life, your functioning at home and at work?
Cultural dimension
What expectations do you have concerning medical care/ your GP? Do you hide your symptoms/ illness from your family, relatives, friends, etc.? If so, why?

The average duration of the interview was 40 min. Some patients responded emotionally to the stories they told and cried or presented other emotional signals such as self-touching during the interview. After switching off the cameras, the patients analysed the videos with the GP. Most patients agreed that combining the interview and video filming helped them to understand the relationship between what they experienced, their emotions and their symptoms. It appears the patients found that the video registration had the additive effect of controlling their own behaviour, including their symptoms. They did not visit their GP for the next 3 months, and did not change the primary care setting.

### Data analysis

The interview data were subsequently transcribed verbatim by two research assistants, verified by one of the authors (AS), and coded using NVivo 10. The method aimed to establish a straight descriptive summary of the informational contents of the data [31, 32]. The transcriptions were analysed independently by the two authors, who came from different disciplinary backgrounds: linguistics and health communication (AS), and primary care (SCz). Firstly, they were read thoroughly in order to obtain a sense of the texts as a whole, and a first-level, open coding was made for contents. Next, the codes were compared and matching codes were grouped into sub-themes and themes to identify key features of the patients’ views. The researchers paid attention to recurring patterns and the words that best captured the patients’ thoughts. The results were discussed by both researchers several times and necessary adjustments were made, to ensure validity. Table 2 demonstrates examples of meaning units, condensed meaning units, interpretation and formulated sub-themes and themes. The quotations representing the themes were next translated by the linguist (AS).

**Table 2:** Example of meaning unit, condensed meaning unit, interpretation, subthemes and themes from content analysis

Meaning Unit	Condensed meaning unit, description close to the text	Interpretation (Code)	Subtheme	Theme
(1) “When I come to the doctor and he asks: ‘How are things?’, I say: ‘All the same’. It’s a situation in which simply nothing gets better. So…so I get medicines again and… and again it all goes on.”	How are things, all the same, nothing gets better, I get medicines again, it all goes on.	Description of the patient’s condition	No improvement no change	Experience of symptoms
(2) “I explain this to myself as follows: all the time I’ve been in an environment where there was a very tense and nervous atmosphere and it affected me...”	I explain this by being in a tense and nervous atmosphere, and it affected me.	The impact of a stressful environment on the patient	External social factors	Explanation of symptoms

## Results

Four major themes were identified in the analysis**:** patients’ experiences of symptoms; coping; patients’ explanations for symptoms; and expectations about healthcare.

### Patients’ experiences of symptoms

Within the first theme, two sub-themes emerged: symptom persistence or variability, and negative emotions. Most patients admitted that their symptoms had changed with time, whereas some patients claimed they were persistent. When the patients perceived the symptoms as the same*,* usually little or no improvement in their condition was reported. As stated by one of the patients:

P15: When I come to the doctor and he asks: ‘How are things?’, I say: ‘All the same’. It’s a situation in which simply nothing gets better. So…so I get medicines again and… and again it all goes on.

When the patients perceived a change in symptoms and their intensity, they often reported a better understanding of their illness. In the following extract, the patient points to a change in symptoms and her ability to control serious physical symptoms she used to suffer from:

GP: Are they [the symptoms, AS] the same (P2: No) and can you control them better?

P2: No. They are different because I used to have many physical symptoms and now, because you have to hide certain things, I don’t allow myself to have physical symptoms, which are visible, but it all goes to the head.

Another patient describes this process in the following way:

P20: I have a feeling that this tension is wandering all over my body, right (…). There is always something, a part of the body that in a given period of time is in focus and I am convinced that it is ill, that there is something wrong with it, because it gives me a lot of pain, there is something weird in it. And then it moves to something else, right? It is changing like this, but I’m never free from this.

Although the patients differed in their experiences and had difficulty describing emotions they felt, most of them disclosed associated negative emotions of fear, anger, powerlessness, and shame. The onset of symptoms was usually accompanied by fear of a serious disease or death, or failure to recover.

GP: ...what did you feel when the symptoms occurred? (…)

P1: When it comes to the tremors in particular, I felt infernal panic because I was convinced that something wrong was going on with me ...

Patients also admitted to experiencing sudden, irrational fears related to their environment:

GP: And because of these symptoms were you unable to do certain things?

P13: Oh yes, it happened. Last time, half a year ago, I came to work and for no obvious reason I got... so to say... I felt fear; I sat at the desk and I started to fear something, I wasn’t able to work, I was close to tears, I bent down and I was terrified of something, but didn't know what.

As shown in the example, fear was often constructed as a permanent emotional state, accompanying physical symptoms:

P15: ...I am a bundle of nerves, I’m afraid of everything, I won’t go out for a walk, I won’t go anywhere, I always need to have someone next to me and this is it. (...) I live in constant fear, I’m constantly thinking something bad is going to happen.

Apart from fear, patients also pointed to powerlessness:

P4: It was also related to a kind of feeling as if I had a blackout, that I was so powerless. I couldn’t get down to anything, couldn’t start doing anything, but I had to simply go to bed, I don’t know, wait till it’s over, but it simply overpowered and stopped me. I couldn’t start doing anything either, but it stopped me from everything (...). It was related to a kind of...a kind of powerlessness, that I stayed in bed, my body was so inert that I felt as if I was just collapsing, that I wasn’t even able to text anyone or hold my mobile phone, but simply... I lay in bed and I was weak with pain*.*

Another emotion reported by the patients was anger. The patients felt angry when the symptoms reappeared, which prevented them from moving on with their everyday routine.

GP: And when it comes to these symptoms (...) how do you react emotionally? What do you feel then?

P6: Damn it, I get angry that it caught me again. And this is a problem because because all the time, whenever it happens to me, it is at work.

Finally, some patients reported they felt ashamed of their symptoms and hid them from others. As explained by one of the patients:

P1: … I didn’t talk to people much unless they were trustworthy because I was kind of ashamed of this...I didn’t want to be Psycho Number 1 in company so not too many people knew.

### Coping

The majority of the investigated patients admitted that they accepted the symptoms and had learnt to cope with them. Few patients reported feeling completely powerless while faced with the symptoms. According to the patients, successful coping strategies include the rationalization of the symptoms and the awareness of their psychological origin, self-development, and ignoring the symptoms.

What the patients found relevant was the moment they had realized that their symptoms were “psychological” and a serious illness was excluded. Only then could they learn to control the symptoms to some extent, and live with them. As one of the patients asserts:

P10: When I know that I’m able to rationally explain to myself that yes, it will pass, it is nothing that really puts my life at risk, that it is something that is probably related to my nerves, to a certain extent I’m able to control the situation (…). It seems to me that what helped me is the awareness, this increased awareness that it is… it has a psychological origin… and I think it is the most important thing, which I think is likely to help me to pull myself together in the future.

Others drew attention to self-development:

P7: I did a lot of self-work and I realized that… that… indeed… that it is not so easy to break me…

Furthermore, ignoring the symptoms or simply focusing attention on something else, for example reading a book, was reported as one of the most successful ways of coping with the symptoms:

P12: Replacing some thought, so that, I don’t know, I can focus on something else.

### Patients’ explanations for symptoms

Most patients drew on various explanatory frameworks while describing their symptoms. Many of these accounts were expressed with uncertainty. Some patients explained their symptoms as a result of external, social factors, such as stress or life events; some admitted that the origin of the symptoms lies within themselves; others mentioned both personal and social factors and a few provided parallel somatic explanations.

An accumulation of stress caused by external factors, for instance, stressful situations at work or personal relations, was reported as one of the main triggers of the symptoms, as indicated in the following example:

P16: I explain it by the fact that, as I say, all the time I was in an environment where there was a very tense and nervous atmosphere, and it affected me…

When patients explained the symptoms as due to personal or internal factors, they mentioned genetics, personality or multiple psychological causes, and sometimes considered their illness as weakness. In the following example, the patient perceives his symptoms as a result of personal weakness and his inability to cope with things in life:

P6: You know what, you know what, I will tell you: I think they are due to my weakness…I have…I have…damn it… I have certain things to resolve, which I have to…I have to resolve and I’ve been putting them off for so terribly long…

In the next example, the patient states that her symptoms are inborn:

P15: I think that I inherited all these things from my mum. (…) because I remember when I was a kid the ambulance often came to her and it may have touched me and it probably grew, it grew…

While explaining the nature of their symptoms, the patients often underlined psychological causes:

GP: Where do you think the symptoms come from?

P14: I think that it all… comes that it… in my head…somewhere…right? it starts, right? Because because… this chest pain… it can’t have started by itself…. It is... when I start to analyse something, think about some situation, or about work, or a slight conflict in a relationship, all these things trigger the symptoms.

Finally, apart from social or personal explanations, a few patients tried to provide parallel somatic explanations, in which they referred to the body’s malfunctioning:

P1: I assume that...still... the whole body, also the brain, including what appears in it in the form of thoughts, are the connected vessels, so it is as if... I would call it, I explain this by some sort of... so to say a theory of impulse... in other words, if a thought is created, there is still a chemical reaction in the brain, right? Thus, if the thought is stressful, something is going on there, already at the level of cells or the hormonal level, so, if it happens in the brain, it may permeate to the rest of the body because because because it is as if... erm... I will say...erm... poisoned underground waters can poison the river, right?

### Expectations about healthcare

The fourth major theme was patients’ expectations about the healthcare system. The majority of the patients admitted to disengaging from medical services or had no expectations, pointing out that extra tests or examinations would not reveal anything new:

GP: Do you seek help with other doctors, tests…?

P11: No. No, because the tests don’t seem to show anything (…). Yesterday I picked up health outcomes, I thought they were not mine – they were so good.

GP: Mhm

P11: So I think no…that.. that… it just doesn’t make sense.

Others were convinced that the best option was to accept the situation and not to probe further, if there is no need. They stressed they visit psychologists or psychiatrists privately:

P10: …I had two choices: either believe in the diagnoses or probe deeper (…) go from doctor to doctor, look for new medical tests, as if – let’s not be afraid to say that – to plunge into hypochondria; or go in the opposite direction and accept that it is not a real disease, I mean an organic disease, but that is kind of something of psychological nature and nothing in reality is wrong with me… I rather chose this way, for half a year I haven’t visited doctors, I visit a psychiatrist instead because I was diagnosed, in fact, with depression and my treatment has this angle, I also go to a psychologist and that’s it. I avoid doctors, if there’s simply no need.

Few patients expected the legitimization of their symptoms and publicizing the problem of MUS in the Polish healthcare system. However, they did wish to be taken seriously, on a par with patients with physical symptoms, and not to be dismissed:

P2: The first, most important issue is the publicizing of this problem because people like me will not seek help (…). I feel that people with such problems are pushed away because these are not physical symptoms. So, there are no screening tests because people like me do not exist… the help should be clearly defined… where I can go, what I can do in such situations, but there isn’t anything like this, so people like me don’t look for help.

## Discussion

This study recognizes the importance of patients’ personal accounts of living with MUS and the necessity of appropriate and acceptable explanation of symptoms. Four major themes were identified: patients’ experiences of living with MUS, patients’ explanations, coping and expectations regarding healthcare.

The main finding of our study is that patients’ acceptance and acknowledgement of the symptoms’ psychological origin may turn out to be empowering for some patients with MUS. Many patients participating in our study highlighted, in their accounts, the moment they had realized that their symptoms were “psychological.” According to the patients, this awareness allowed them to learn to live with the symptoms and to some extent control them. The patients who perceived a change in symptoms, and their intensity, often reported a better understanding of their illness than those for whom the symptoms were the same. A few patients in our study felt completely powerless about MUS, and told chaotic narratives.

This suggests that experiences of living with MUS might differ depending on the state of acceptance of illness, the level of insight the patients gained, as well as on the stage they are at, in the diagnostic process. As pointed out by Dwamena et al., “MUS patients who endorse psychological explanations and insights may have better coping mechanisms, and may be the most propitious group for treatment that emphasizes plausible explanations that are acceptable to the patient” [7].

This is in contrast with previous studies conducted by Nettleton et al., which demonstrated that, for some patients with MUS, a psychological explanation may be a confirmation that the symptoms are not “genuine” and, thus, their illness is “illegitimate” [13]. Likewise, Kornelson et al. argue that a psychological explanation for unexplained symptoms is not legitimate for everyone, as it often entails a social stigma [17]. Kornelsen et al. observed that psychologizing the patients’ symptoms “implied to them that they should be able to ‘fix’ themselves somehow” [17]. In other words, acceptance of psychological explanations may, at the same time, put the patients in a position where they are socially expected to help themselves, and not to look for support or utilize medical services. Our study partially confirms this: some patients perceived the management of the symptoms as their own responsibility, engaged in self-work, ignored the symptoms, or sought help from private psychologists and psychiatrists.

Overall, while explaining their symptoms, our respondents drew on various frameworks, focusing on psychological and social causes, which are frequently ignored in clinical settings [33], rather than providing a somatic explanation [17–19, 21, 22]. If the patients explained the symptoms as a result of personal weakness or an inborn defect, these accounts were usually accompanied by a feeling of shame or reported self-blame. In contrast to other studies by Lidén et al. [19], or Nunes et al. [34], some patients admitted they had not spoken much to other people about their symptoms, or had deliberately hidden the symptoms. Shame and stigma often accompany illness [24]. However, as observed by Kleinman, the stigma begins with the patient’s own acceptance of a stigmatized identity. The patient tends to feel shame not because of cultural meaning attached to the illness label, but rather in response to the reactions of health professionals or family members [35]. The study by Freidl et al. [23] corroborated anticipated stigmatization among patients, in particular patients with somatoform pain disorder.

Our study also reveals the necessity to raise discussion of the problems of patients with MUS in the Polish healthcare system. What might seem surprising is the patients’ lack of expectations in this regard. In our study, only a few patients expected that their symptoms would be treated on a par with physical symptoms by healthcare practitioners, while the majority did not expect medical services to help them improve their condition, stating that they do not visit doctors, apart from their GP. Although this seems to contradict a common perception of patients with MUS as high utilisers, it may be explained by the patients in our sample, who have already been through a long diagnostic and treatment process, and are thus at a later stage of their illness [4, 5]. Another possible explanation is stigmatization of patients with MUS in Polish primary care, which our previous studies confirmed [26, 27]. According to Stone, “[s]ome patients quietly disengage from health services altogether” if they “can't face engaging in a process that invalidates their pain” [24]. The legitimatization of symptoms is one of the most widely recommended strategies for patients presenting MUS, and should also be recognized by Polish healthcare practitioners.

Another important finding of our study is that the patients frequently admitted to undergoing psychotherapy or visiting psychologists privately. This can be explained through the specifics of Polish healthcare, where access to psychologists and psychiatrists is restricted and there are long waiting lists for specialist treatment, which discourage patients with MUS from seeking support in the public sector. In the light of this barrier, the role of the GP in helping patients to interpret and learn to live with MUS is crucial.

Our study acknowledges the importance of the therapeutic relationship and a personalized approach, which implies provision for the patient’s needs, concerns and emotions. In our study, MUS patients disclosed negative emotions associated with the symptoms: fear, powerlessness, anger and shame. When patients conceptualized fear as a permanent state, their accounts occasionally sounded like descriptions of an anxiety disorder, which might possibly be due to the strong syndrome overlap [36]. Generally, patients with MUS are perceived as emotionally inhibited. One of the theories explaining MUS posits that somatic symptoms result from unresolved emotional conflicts and are associated with repression and overcontrol of emotional expression [12]. Patients with MUS can be offered a filmed interview combined with a follow-up analysis with their GP to better understand their symptoms, associated emotions and nonverbal behaviour.

### Strengths and limitations

This is a unique qualitative study exploring Polish patients’ perspectives on living with MUS. A qualitative approach is one of the best methods to elicit patients’ accounts and gain a deep understanding of their illness experiences. Our study extends knowledge of patients’ experiences with MUS. However, some limitations should also be considered. The patients in our study were recruited from a single GP’s practice, and most of them were interviewed by their own GP. This may have introduced a bias in the sample, reflecting experiences from a selected patient group and a special doctor-patient relationship. On the other hand, this allowed us to explore personal and sensitive themes and elicit accounts, that were often intimate, of the patients’ experiences of MUS, which they would not have revealed to anyone else. It should also be noted that all patients in our study were diagnosed with F45, thus were at a later stage of their illness, which may have restricted the variations in our findings. However, the selection of the group seems justified and shows that experiences of living with MUS differ depending on the stage of the diagnostic and treatment process and the level of insight gained by the patients.

## Conclusion

This is the first study that reports on Polish patients’ perspectives on living with MUS. Improving the management of patients with MUS in primary care requires the allocation of increased financial resources for GP training and facilitated access to psychologists and psychotherapists. Future research should be extended to other primary care settings, and should focus on addressing these patients’ psychological needs and concerns during consultation between GPs and these patients. One of the options could be a filmed consultation with a follow-up analysis with the GP as a potentially effective and additive strategy of MUS management, especially in a long-term perspective.

## Abbreviations

DSQ: Four-Dimensional Symptom Questionnaire
GP: General practitioner
ICD: International Classification of Diseases
MUS: Medically unexplained symptoms
